# Microfluidic preparation of monodisperse polymeric microspheres coated with silica nanoparticles

**DOI:** 10.1038/s41598-018-26829-z

**Published:** 2018-06-04

**Authors:** Dong-Yeong Kim, Si Hyung Jin, Seong-Geun Jeong, Byungjin Lee, Kyoung-Ku Kang, Chang-Soo Lee

**Affiliations:** Department of Chemical Engineering and Applied Chemistry, Chungnam National University, 99 Daehak-ro, Yuseong-gu, Daejeon, 34134 Republic of Korea

## Abstract

The synthesis of organic-inorganic hybrid particles with highly controlled particle sizes in the micrometer range is a major challenge in many areas of research. Conventional methods are limited for nanometer-scale fabrication because of the difficulty in controlling the size. In this study, we present a microfluidic method for the preparation of organic-inorganic hybrid microparticles with poly (1,10-decanediol dimethacrylate-co-trimethoxysillyl propyl methacrylate) (P (DDMA-co-TPM)) as the core and silica nanoparticles as the shell. In this approach, the droplet-based microfluidic method combined with *in situ* photopolymerization produces highly monodisperse organic microparticles of P (DDMA-co-TPM) in a simple manner, and the silica nanoparticles gradually grow on the surface of the microparticles prepared via hydrolysis and condensation of tetraethoxysilane (TEOS) in a basic ammonium hydroxide medium without additional surface treatment. This approach leads to a reduction in the number of processes and allows drastically improved size uniformity compared to conventional methods. The morphology, composition, and structure of the hybrid microparticles are analyzed by SEM, TEM, FT-IR, EDS, and XPS, respectively. The results indicate the inorganic shell of the hybrid particles consists of SiO_2_ nanoparticles of approximately 60 nm. Finally, we experimentally describe the formation mechanism of a silica-coating layer on the organic surface of polymeric core particles.

## Introduction

Organic-inorganic hybrid materials have been extensively investigated for a long time. They are generally organic polymers incorporated with inorganic nanoscale building blocks. The combination of the inorganic material with the organic polymer provides complementary properties, such as high rigidity and flexibility, thermal stability and processability, and ductility. A distinctive feature of organic-inorganic hybrid materials is that the small size of the nanoparticles leads to a dramatic increase in the interfacial area compared with traditional materials or composites. This interfacial area creates a significant volume fraction of the interfacial polymer with properties different from the bulk polymer even at low loadings^1^. Recently, there are many considerable efforts to synthesize organic-inorganic hybrid particles because they can provide remarkable properties by the appropriate combination and structuring of the organic and inorganic materials^2,3^. For example, a wide variety of colloidal inorganic materials has been used for polymer-based hybrid particles, including titanium dioxide^4,5^, iron oxide^6^, zinc oxide^7^, clay^8^, sliver^9,10^ and gold^11,12^. Among the many inorganic coating materials, silica particles are one of the most interesting candidates since they are widely used in many fields, such as colloidal and material science and engineering, and they exhibit special properties, such as biocompatibility, low toxicity, and scalable synthetic availability, that are substantially different from those of bulk material.

Furthermore, the incorporation of silica particles into nanocomposite colloids offers many promising perspectives. Thus, hybrid particles with a core-shell structure are attracting a great deal of interest because of their diverse applications as catalysts^13^, protective coatings^14^, and drug delivery materials^15^. In addition, the physical features of the hybrid particles with core-shell structure provides a high surface area, controllable morphology and roughness^16–19^, protection and encapsulation of the core materials, and the promising conjugation moieties for a variety of chemical and biological molecules^20–26^.

Efforts for the preparation of organic-inorganic hybrid particles based on the silica coating on the core materials have been reported^27–29^. However, there are several problems, including (1) the limitation on the synthesis of core-shell microparticles. For example, the formation of the pore structure in the shell induces a decrease in the mechanical strength of the shell and enhances the diffusion of molecules from environment into or through the coated particles, which can deteriorate the stability of the precious core material inside the core-shell particles and finally change the properties of the materials; (2) the incomplete or inhomogeneous coating of silica particles. The organic-inorganic hybrid structure is important because this structure can increase the shell strength and avoid the penetration of larger molecules into or out of the particles^30^. A thin shell (thickness <60 nm) with a nanometer-scale, homogenous thickness and a smooth surface is produced, while in the current reports, the modification of the shell in this thickness range (when using a submicron to micron size core) is typically difficult and relates to the formation of an incomplete/inhomogeneous shell and a rough surface; (3) the lack of detailed information about the control of shell thickness, shell structure, and agglomeration phenomenon; (4) the limitation on the production of highly monodisperse hybrid microparticles with controllable manner; (5) the control of the size of core and shell particles.

To overcome the above problems, the surface of organic core particle should have sufficient affinity for inorganic silica precursor. Several approaches have been proposed for increasing the affinity between the core and the shell components. For example, layer-by-layer (LBL) assembly through sequential adsorption of polyelectrolytes (PELs) has been utilized to generate electrostatic attraction^31–33^. Surface modification based on the treatment of the amphiphilic coupling agent with preformed polymer particles are demonstrated to increase chemical affinity through van der Waals force, hydrogen bonding and dipole-dipole interaction^34–36^. Additionally, the synthetic procedures commonly require significant affinity between the inorganic silica particles and polymers to circumvent their inherent incompatibility.

However, the synthetic procedures are generally complicated and require multistep processing despite the success of coating silica particles onto the polymeric particles. When using silica nanoparticles as a silica source, we obtain the inhomogeneous shell thickness, the surface roughness, and the existence of porous structures in the final hybrid particles. Thus, an elegant strategy to establish a physicochemical or chemical link at the interface of the organic and inorganic constituents is an inevitable issue.

In general, polymeric microparticles as a core part are synthesized by emulsion polymerization, which utilizes a mechanical agitator to provide shear stress between two immiscible fluids. The provided shear stress forms an emulsion that can be polymerized into polymeric microparticles. However, one drawback consists in the rather broad size distribution of the polymeric microparticles. Microfluidics has emerged as one of the most attractive technologies for producing many polymeric materials that are useful for materials science and engineering, foods science, pharmaceutical technology, and biotechnology^37,38^. Microfluidic approaches offer finely controlled environments for producing monodisperse emulsions, which are considered to be the most reliable alternatives to the conventional bulk emulsification method.

Furthermore, microfluidic approaches based on controlling the hydrodynamic parameters have enabled the formation of nonspherical emulsions within confined microfluidic geometries such as disks, rods, and ellipsoids, resulting in the production of asymmetric particles^39,40^.

Here, we present the microfluidic preparation of monodisperse polymeric microparticles coated with silica nanoparticles as organic-inorganic hybrid particles without an additional surface modification step. The polymeric microparticles prepared with various sizes (ranging from 10 to 300 μm) are used as a model core organic material. Owing to the flexibility offered by the droplet-based microfluidic approach, the monodispersity, morphology, and size can be modulated by simple manner. As a result, new types of hierarchical organic–inorganic hybrid particles consisting of silica nanoparticles layered structures on the micron sized organic materials have been obtained. In contrast to previous silica-coating approaches, the polymeric core particles are synthesized through *in situ* microfluidic incorporation of 3-(trimethoxysilyl) propyl methacrylate (TPM) to contain silanol groups on their surface, which can act as functional moieties on the surface of polymer particles, thus enhancing their affinity to silica nanoparticles or silane molecules. This combined approach allows for the creation of a silica coating without an additional surface modification or re-nucleation step.

Finally, the investigation of the formation of the silica shell is also presented along with the theoretical consideration and proposal of the silica shell formation mechanism that forms a fully covered surface grown on the surface of polymeric core microparticle (forming core-shell hybrid particles); this information is typically disregarded in the current silica coating reports.

## Results and Discussion

**Figure 1:**
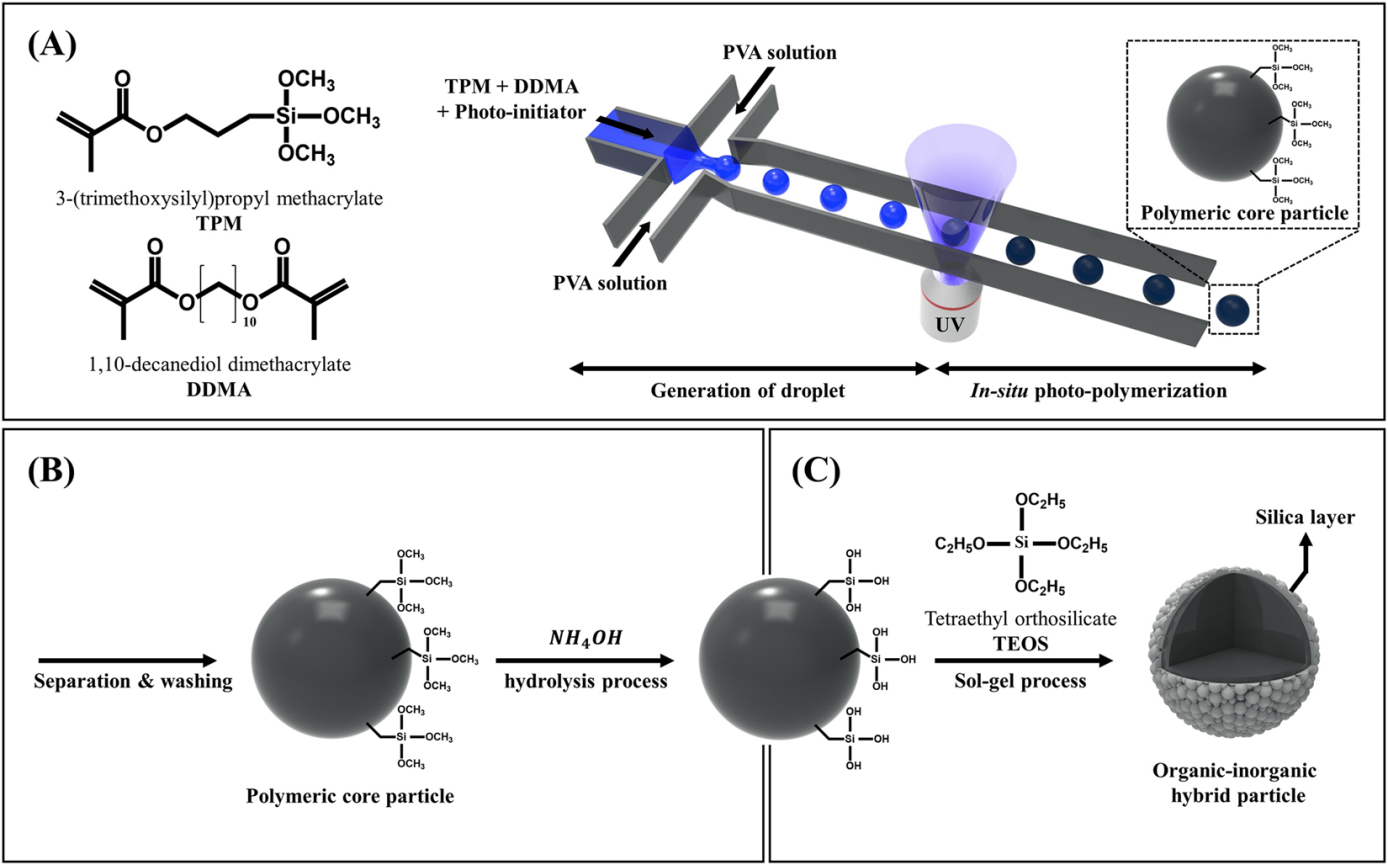
Schematic illustration of the preparation of monodisperse hybrid particles (organic microparticles coated with inorganic silica nanoparticles). (**A**) Photocurable droplets containing the monomers (TPM and DDMA) and the photoinitiator in the continuous phase (PVA solution) are formed by a shear force driven pinch-off mechanism. Under UV-irradiation, photopolymerization is initiated. (**B**) Hydrolysis of ehtoxide group for the growth reaction of silica particles onto the polymeric microparticles. (**C**) Final stage of complete synthesis of polymeric core microparticle coated with silica nanoparticles as a shell (organic-inorganic hybrid particle).

The schematic synthesis procedure of organic-inorganic hybrid particles is shown in Fig. 1 in which the microfluidic method is used for the preparation of highly monodisperse polymeric core particles, and a subsequent sol-gel reaction is performed.

In the microfluidic synthetic step, two immiscible fluids are used to generate photocurable droplets. Here, a disperse fluid consists of 1,10-decanediol dimethacrylate (DDMA) (polymeric core) and TPM (functional moiety for the chemical linking of DDMA and tetraethyl orthosilicate (TEOS)), and the mixture of polyvinyl alcohol (PVA) and water acts as a continuous fluid (Fig. 1A). Chemically, the TPM has two interesting functional moieties; the acrylate and silane group in the TPM can form covalent bonds with DDMA under photopolymerization and with TEOS in the sol-gel reaction, respectively. First, we create discrete droplets in a flow-focusing geometry when two immiscible fluids are introduced (Fig. 1A). We illuminate the drops with UV light to polymerize DDMA and TPM. This photoploymerization enables rapid production of monodisperse particles at room temperature^39,41,42^.

Next, for the efficient synthesis of the silica nanoparticle shell, TPM is subsequently hydrolyzed by replacing the existing ethoxide groups with hydroxyl groups on the surface of the polymeric core particles, forming activated silanol groups (Si(OH)_3_) (Fig. 1B). It is an important step to induce the generation of a silica seed on the surface of the polymeric core particles at the beginning of the sol-gel process.

After the TEOS solution is added into the aqueous solution, most of the TEOS is decomposed into silicic acid (Si(OH)_4_), which is an important silica-forming monomer. The silica monomers coalesce, condense, and polymerize to form nuclei. The nucleus is the smallest size of silica, which has a strong affinity to the activated silanol groups (Si(OH)_3_) on the surface of the polymeric core particles (Fig. 1C). Finally, the attached nuclei attract the silica-forming monomers in the medium to form the silica nanoparticle shell on the surface of the polymeric core particles.

### Generation of monodisperse droplets

**Figure 2:**
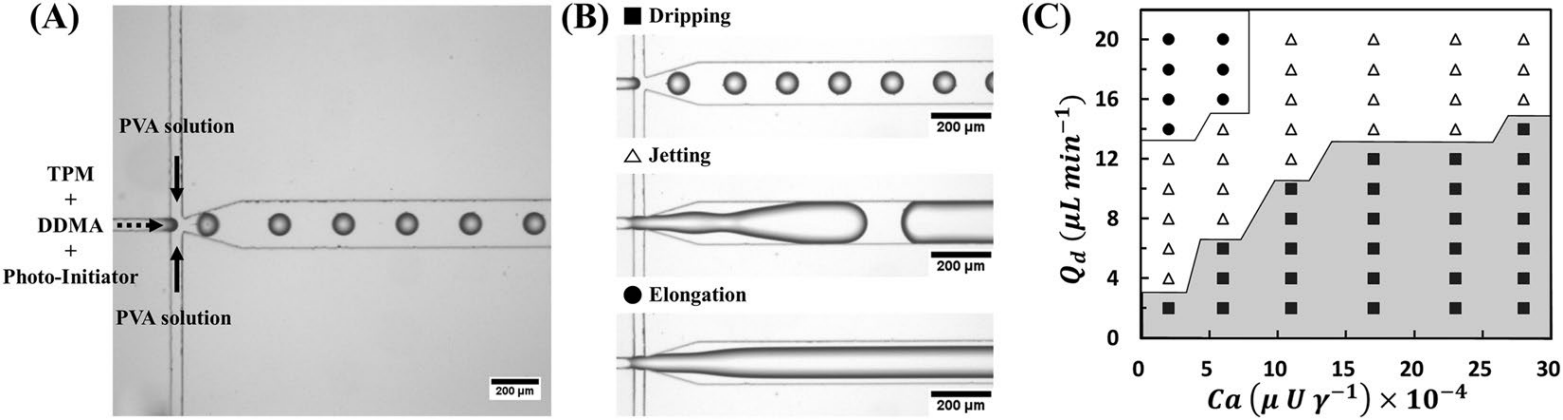
Generation of droplets and the flow patterns observed in the microfluidic device. (**A**) Optical microscope image of droplets in the flow-focusing microchannel. Black and dashed arrows represent continuous and dispersed fluid flow, respectively. (**B**) Optical images of three different flow patterns in the flow-focusing junction. (■) dripping, (△) jetting, (●) elongation. (**C**) Phase diagram as a function of the flow rate of the disperse phase (Q_d_) and the capillary number (*Ca*) for three different flow patterns. Phase diagram shows three representative flow patterns.

To prepare highly monodisperse polymeric core particles, it is essential to generate uniform droplets in microfluidic device before performing the photopolymerization process. The microfluidic system consists of the dispersed phase containing photo-curable monomers (DDMA and TPM) and photoinitiator (Darocur 1173), and the continuous phase with an immiscible PVA solution (Figs 1 and 2). The photo-curable dispersed phase is injected from a center channel and the continuous phase (aqueous PVA solution) that is to surround the droplet is introduced from two side channels. These fluids flow into cross-flowing streams, where shear and pressure fluctuations generated by two counter flows squeeze the dispersed phase, producing droplets (Fig. 2A). We selected PVA solution as a continuous phase to prevent the merging of droplets generated because it is immiscible and does not react with photo-curable droplets.

The flow streams become unstable and consequently the photo-curable dispersed phase breaks up into droplets in the downstream of the microchannel because the aqueous continuous phase perfectly wraps dispersed droplets (Fig. 2A). When the two phases come into contact at the channel junction, symmetric shear forces from the continuous phase are generated on the dispersed phase, enabling the consistent separation of droplets with uniform size. At this point, the continuous phase begins to wrap around the dispersed phase due to the difference in wettability between the two fluids with the PDMS wall, which has a hydrophilic surface property treated with an oxygen plasma.

A force balance between the viscous stress and the surface tension stress determines the formation of the droplets at the junctions^43–45^. The dimensionless capillary number (*Ca* = *Uμ*/*γ*) represents the relative force balance acting an interface between two immiscible fluids, where *U* indicates the linear flow rate of continuous phase, *μ* represents the viscosity of the continuous phase, and γ is the interfacial tension between the dispersed and continuous phases. *Ca* is controlled by manipulating Q_c_, the flow rate of the continuous phase, leading to a variation in *U* based on the relationship *U* = Q_c_/A, where A is the area of cross-section of the microfluidic channel. In general, a phase diagram indicating different flow patterns observed as a function of the experimental factors is an important information for understanding droplet formation^46,47^. As shown in Fig. 2C, the flow patterns of our system as *Ca* and Q_d_ coordinates clearly indicates an important regimes for the formation of monodisperse droplets. We observe three typical flow patterns in the junction such as the dripping for the formation of stable droplets, jetting with unstable droplets, and an elongation flow as shown in Fig. 2B and C.

Monodisperse droplets are generated only within a limited regime where the flow rate of disperse phase is at least 2 μl/min. As the flow rate of the dispersed phase increases, the *Ca* range required for stable droplet formation is limited, which reduce the stable droplet generation range. Thus, a dispersion phase at a high flow rate cannot produce monodisperse droplets. As *Ca* decreases gradually from a constant value of Q_d_, it reaches a critical capillary number that begin to break into droplets. This is the beginning of the transition from a stable droplet regime to an unstable droplet regime. The further increase in the flow rate of disperse phase (Q_d_) results in the formation of long thread (elongation flow) at above 14 μl/min or unstable droplet formation (jetting) in the range of 4 and 14 μl/min. The droplet is changed into a cylindrical long thread along the microchannel at high flow rates of the disperse phase (Q_d_ > 14 μl/min) because the shear force of the continuous PVA phase at the junction is not sufficient to break off the disperse phase into droplets. In addition, this elongated thread can cause blocking problems within the microchannel due to the increased area and time of photopolymerization.

Therefore, we can clearly distinguish three types of flow pattern. Monodisperse droplets are stably generated with a reproducible size in the dripping regime while unstable droplets and elongated thread are consistently formed in the jetting and elongation regime, respectively. In dripping regime for forming stable droplet, the PVA solution wraps the disperse droplets because of the difference in wettabilities of the two fluids. Thus, we could generate monodisperse droplet-containing DDMA and TPM only in the dripping regime.

### Characterization of monodisperse polymeric core particles

**Figure 3:**
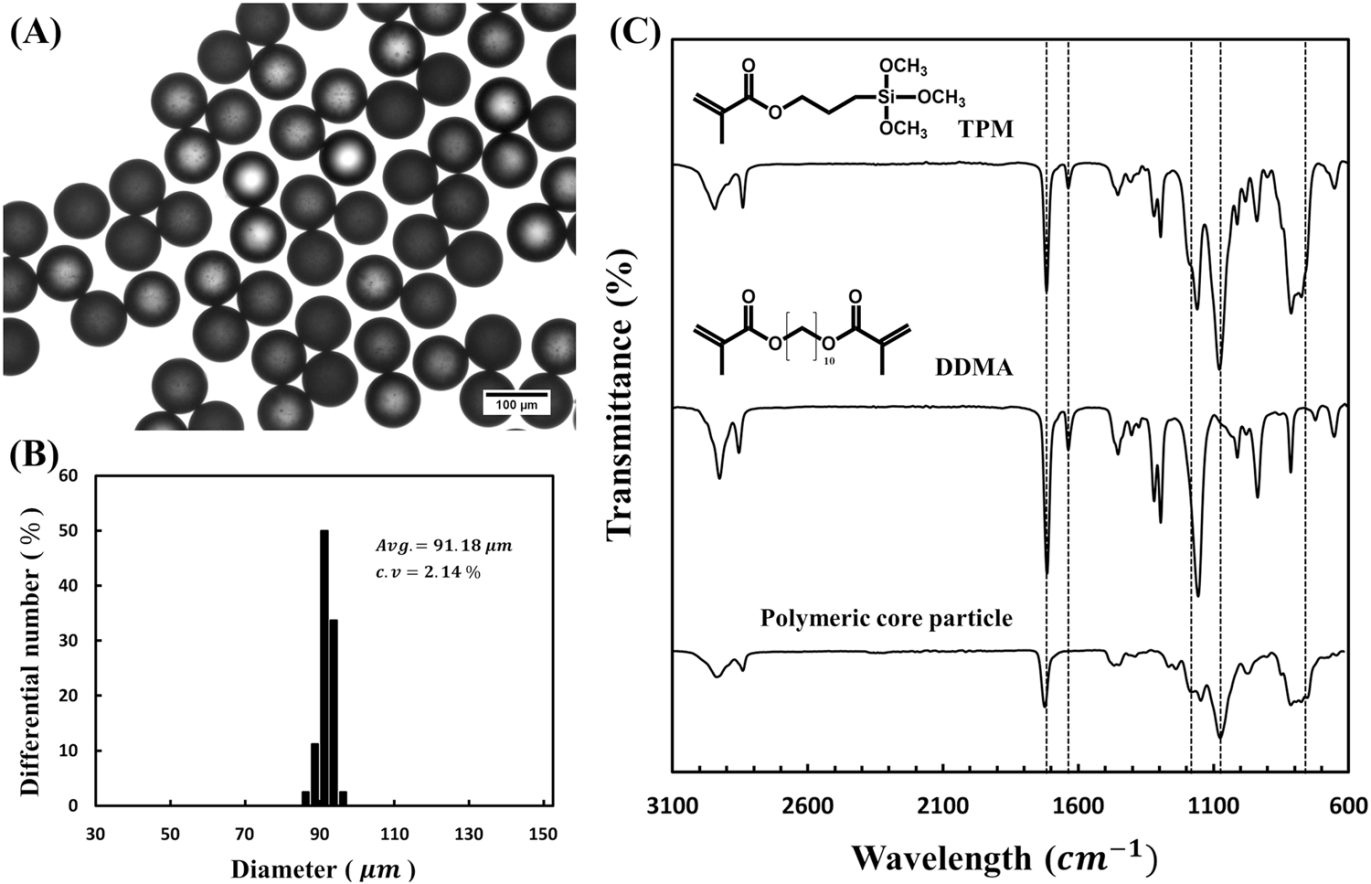
Optical image, size distribution, and FT-IR spectra of polymeric core particles. (**A**) Optical microscope image of the polymeric core particles generated by *in situ* photopolymerization in the microfluidic device. (**B**) Histograms of the size distribution of polymeric core particles with small variation (CV = 2.14%). (**C**) FT-IR spectra of 3-(Trimethoxysilyl)propyl methacrylate (TPM), 1,10-decanediol dimethacrylate (DDMA), and polymeric core particles.

The mechanical stability of polymer core particles prepared using *in situ* microfluidic synthesis is analyzed after sequential washing with two solvents (ethanol and water) and drying thoroughly in a convection oven. As shown in Fig. 3A, there is no microparticle aggregation, and the shape of the microparticles is preserved in the dried status after serious washing with two different solvents. This result clearly shows that *in situ* photopolymerization in the microfluidic device is successfully performed to produce stable microparticles with high monodispersity while incomplete polymerization induces collapse or aggregation of microparticles.

The microparticles produced show narrow distribution of size with an average diameter of 91.2 μm and a coefficient of variance (CV) of about 2.1% (Fig. 3B). This result indicates that the narrow size distribution with a CV < 5% agrees well with the US National Institute of Standards and Technology (NIST) definition of monodispersity^39,48^. Therefore, *in situ* microfluidic method efficiently synthesizes monodisperse microparticles although other microfluidic approaches for the generation of hydrogels or microparticles mainly depend on a post-polymerization processes^42^.

Attenuated Total Reflection Fourier Transform IR (ATR-FTIR) spectroscopy is used to analyze specific chemical bonds of microparticles. Figure 3C presents the FT-IR spectra of the TPM, DDMA monomer, and the microparticles synthesized in the microfluidic device.

The characteristic absorbance peaks of TPM and DDMA are indicated with numbers and arrows. The TPM and DDMA monomer have two distinct absorbance peaks at 1720 and 1620 cm^−1^, indicating the vibration of the acryl group. In particular, the band at 1620 cm^−1^ originates from the C=C double bond^49^. However, the disappearance of the characteristic peak at 1620 cm^−1^ in the FT-IR spectra directly confirm that the dispersed droplets containing photo-curable monomers are successfully polymerized in the microfluidic device. Additionally, the shift of the absorption band from 1720 cm^−1^ to 1740 cm^−1^ is evidence of the polymerization of the acrylate functional group^50^.

Also, we find two characteristic peaks of silicon ethoxide group in TPM at 1165 and 1075 cm^−1^ and the broad absorption peak of Si-C stretching at 700–800 cm^−1^ ^51,52^. These characteristic peaks in the FT-IR spectra are direct evidence of the successful synthesis of microparticles.

FT-IR spectroscopy has been also widely applied to investigate of silica materials^53^. Analysis of FT-IR spectra provides an important indication of the influence of processing parameters on particle’s structure.

The typical FT-IR spectra of the organic core particle obtained before and after hydrolysis process are shown in Fig. S1. While two particles (before and after hydrolysis) have almost similar pattern in the below 3200 cm^−1^ region, apparently, hydrolyzed core particles show characteristic absorbance peak at 3730, 3625, and 3430 cm^−1^. The observed signals at 3730 and 3625 cm^−1^ are ascribed to free silanol groups (Si-OH) stretching and the broad absorption in the region of 3430 cm^−1^ indicates the hydrated silanol groups (Si-OH) on the surface of the particles^54–56^. The difference of characteristic peaks directly prove that polymeric core particles prepared by microfluidic system is not activated as free silanol groups on the surface before hydrolysis process. Also, this result indicates that the droplet composed of TPM and DDMA monomers is polymerized in a short time and there is no catalyst in the water used as a continuous phase in the microfluidic system.

### Formation process of inorganic silica shell

**Figure 4:**
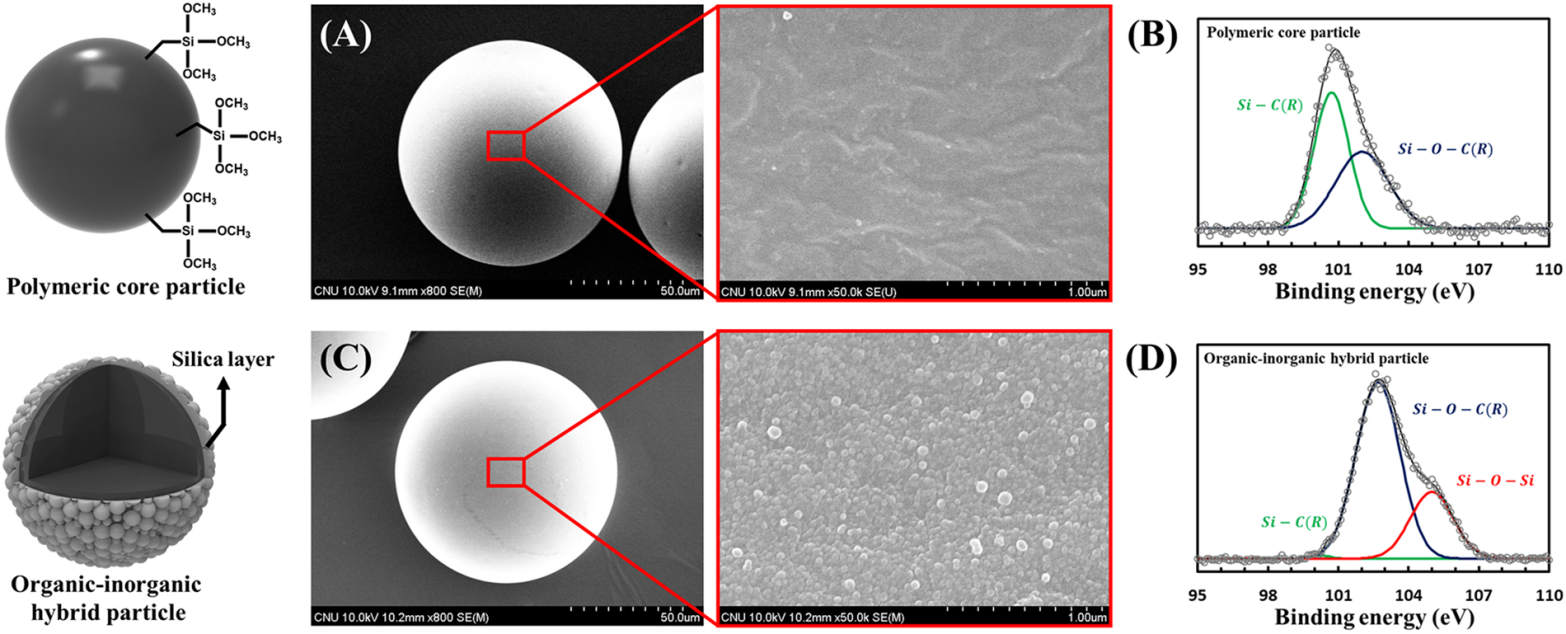
Analysis of the surface morphology and chemical bonding of the polymeric core particles and the organic-inorganic hybrid particles. Left diagrams in the SEM image indicate the illustration of the prepared polymeric core particles and the organic-inorganic hybrid particles, respectively. (A) SEM images and (B) XPS spectra of polymeric core particle composed of TPM and DDMA. (C) SEM images and (D) XPS spectra of organic-inorganic hybrid particles after the sol-gel reaction. The enlarged images indicate the surface morphology and shape of the silica nanoparticles formed onto the polymeric core microparticles. XPS analysis of Si(2p) high-resolution multiplex spectrum collected from (**B**) the polymeric core particles and (**D**) the organic-inorganic hybrid particles.

To investigate the structural details of the microparticles, scanning electron microscopy (SEM) and X-ray photoelectron spectroscopy (XPS) analysis of organic and organic-inorganic hybrid particles are performed to confirm the morphology and chemical bonding (Fig. 4). The structure of the organic-inorganic hybrid particles with a thin silica layer is clearly shown (Fig. 4C). The SEM images show that the shape of both particles is perfectly spherical, and their sizes are almost identical; however, these two particles show a large difference in their surface morphology. In the case of the polymer core particles, no other substances deposited were observed, and the surface of the polymer particles after the sol-gel synthesis was completely covered with the silica nanoparticles. These different surface morphologies are direct evidence that the sol-gel reaction results in the incorporation and growth of silica nanoparticles on the surface of the polymeric core particles. The size of the silica nanoparticles coated on the surface is less than 100 nm. The result of the SEM analysis indicates that the presence of silanol group derived from silicon ethoxide group acts as a silica seed on the surface of the polymeric core particle through a hydrolysis step.

A complementary XPS analysis is used to confirm for the chemical structural information. The survey spectra obtained from both particles prepared reveal the type of chemical bonds formed with the Si element^57–59^. In the deconvoluted high-resolution Si (2p) spectrum of the polymeric core particles, two strong peaks corresponding to Si-C (chemical bond of TPM) and Si-O-C (the silicon ethoxide group in the TPM) are observed (Fig. 4B). The XPS spectrum after the silica-coating process shows three strong peaks at Si-C, Si-O-C, and Si-O-Si (Fig. 4D).

Only difference in the XPS spectra is the existence of a Si-O-Si peak between polymeric core and the hybrid particles, which is strong and direct evidence of the hydrolysis of TPM and the sequential condensation reaction of Si-OH obtained with the hydrolysis of TPM. The result of the condensation is Si-O-Si. However, the peaks could not be observed in the XPS spectra of polymeric core particles, as shown in Fig. 4B and D.

This result confirms that polymeric core particles are only synthesized with TPM and DDMA through *in situ* photopolymerization in the microfluidic system (Fig. 4A and B). In the case of the organic-inorganic hybrid particles, the XPS spectra shifted toward a higher binding energy (Fig. 4D), as in the corresponding SEM images of Fig. 4C, and the decrease in the peak of the Si-C bond obtained from TPM proves that the Si-O-Si bond originates from the silica nanoparticles coated on the surface of the polymeric core particles.

**Figure 5:**
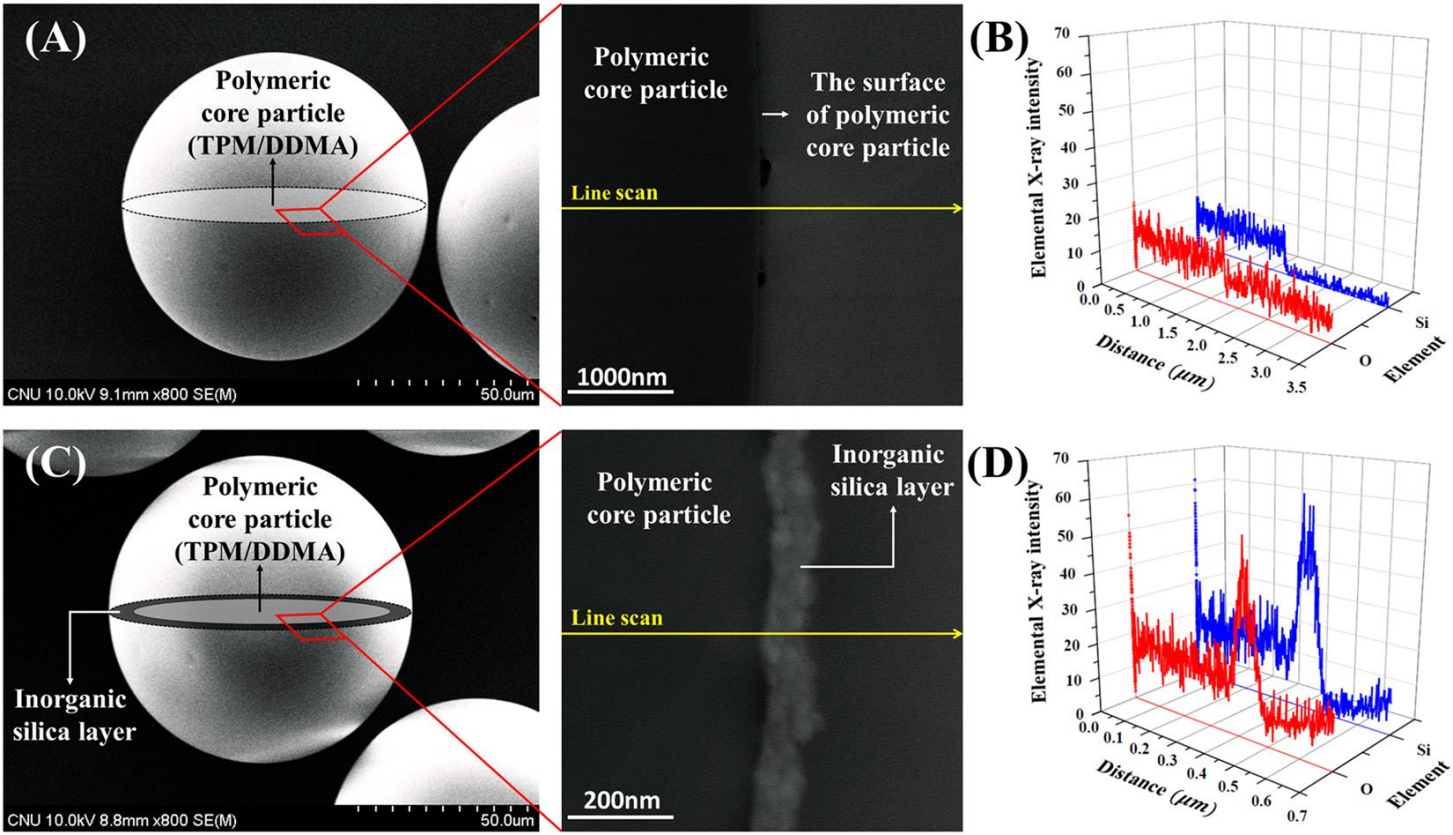
Analysis of the core-shell structure in the particles. (**A**) SEM and TEM image of the polymeric core microparticles before the sol-gel reaction and (**B**) the corresponding line scanning profile of EDS along the cross section of the particles; (**C**) SEM and TEM image of the organic-inorganic hybrid particles after the sol-gel reaction and (**D**) the corresponding line scanning profile of EDS along the cross section of the particles. The SEM images are combined with the schematic illustrations of the expected structure. The enlarged TEM image of the cross section of the particle clearly indicates the existence of a shell layer. In the analysis of the line scanning profile of EDS, the blue and red line represent silicon and oxygen atoms, respectively.

Next, we reconfirm the core-shell structure through direct observation of the cross section of both the polymeric core particles and the organic-inorganic hybrid particles in the SEM, transmission electron microscopy (TEM), and energy-dispersive X-ray spectroscopy (EDS) (Fig. 5) analysis. To achieve a clear image, the particles are sliced to an ultrathin cross section by using ultra-microtoming equipment. The ultrathin sliced samples are approximately 70 nm thick and are investigated by using the high spatial resolution TEM. The enlarged TEM images show that the shell of the organic-inorganic hybrid particle looks like a bright layer because of the difference in the electron density (Fig. 5C). However, in case of the polymeric core particles, there is no clear bright portion (Fig. 5A). The bright colored layer, believed to be a silica layer, is approximately 100 nm.

For more precise investigation of the composition of the particle cross section, the elemental distribution of silicon and oxygen is investigated by using STEM-EDS elemental mapping analysis (Fig. 5B and D). They clearly differentiate the core-shell structure of the as-prepared composites. The Si and O elements are localized in a narrow region, which is consistent with the SEM and TEM results mentioned. As expected, the analysis of the elemental distribution shows that the signals of Si and O elements of the polymeric core particle demonstrate almost constant intensity in the entire region, while the elemental intensity of the organic-inorganic hybrid particle increases extremely at a specific region estimated as the silica shell.

An interesting silica-coating process is attained from a mixing organic polymeric core particles and silica source (TEOS) in the aqueous solution. The process is conducted using neither an additional surfactant nor polymer, which implies that the fundamental reason for the success of the silica-coating process is purely due to the attraction phenomena between the silica and the core components.

**Figure 6:**
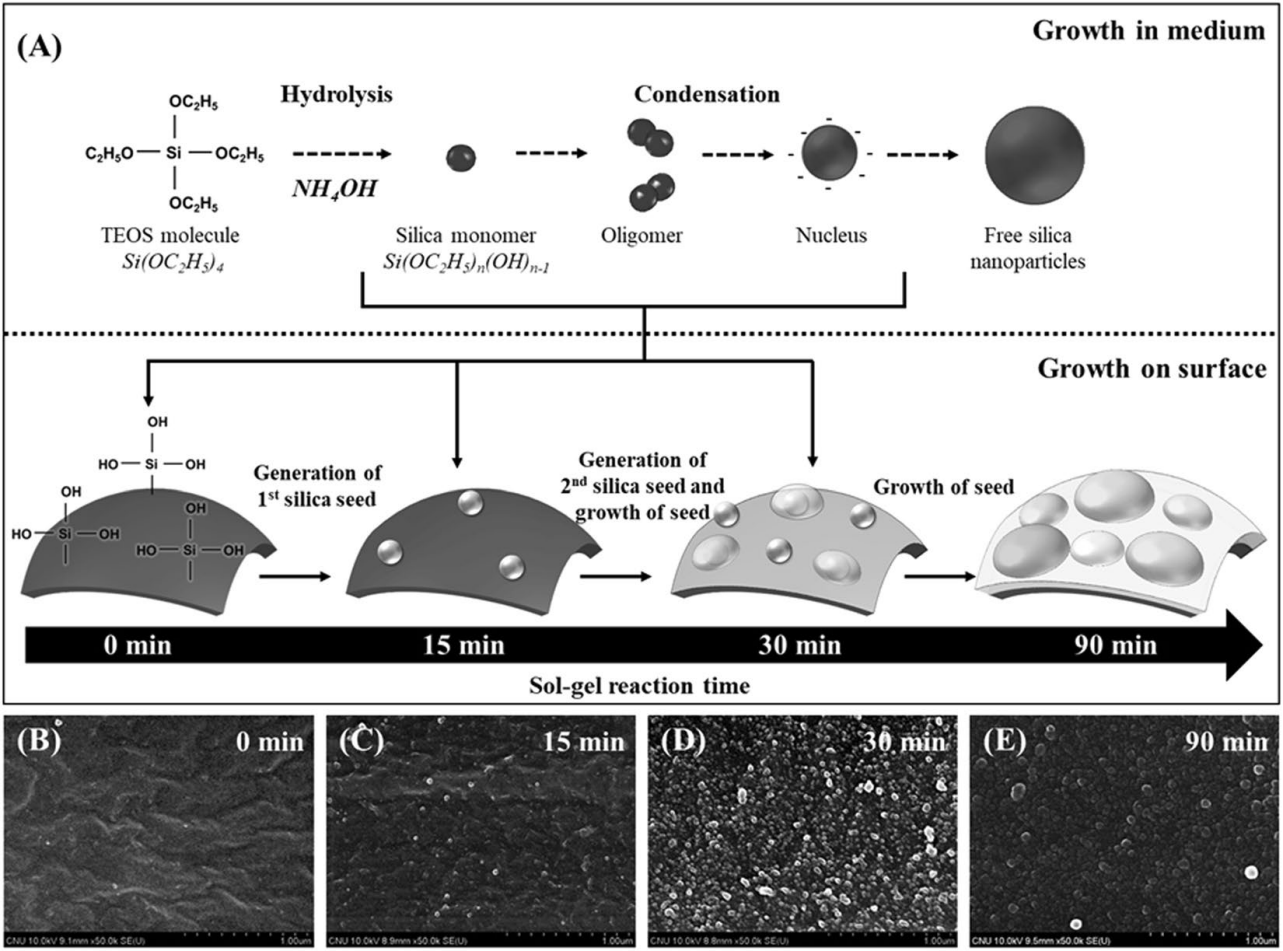
SEM images and corresponding illustration of the mechanism of the formation of the silica coating. (**A**) Schematic diagram for the mechanism of the formation of the silica coating. (**B**) 0 min, (**C**) 15 min, (**D**) 30 min, and (**E**) 90 min. All images are under the same magnification.

Based on the observation of the formation of the silica shell on the surface of the polymeric core particles, we are able to describe the mechanism of formation of the silica shell (Figs 6, S2 and S3). During the hydrolysis step using aqueous ammonia, silicon ethoxide groups are changed into silanol groups on the surface of the polymeric core particles (Figs 1B and 6A).

When the TEOS solution is added, most of the TEOS is dispersed as droplets in the aqueous solution and broken with rigorous mixing to form smaller droplets. The TEOS is subsequently hydrolyzed by replacing their ethoxide groups (OC_2_H_5_) with hydroxyl groups, forming silica monomers (i.e., silicic acid (Si(OH)_4_)^60^. The silica monomers coalesce, condense, and polymerize to form nuclei. The nucleus is the smallest size of silica, and it is attracted by the polymeric core particle^60,61^. Simultaneously, the condensation reaction occurs between the silanol groups on the surfaces of core particle and the silica monomer hydrolyzed from TEOS.

The generation of the seed particles on the surface of the polymeric core particles proceeds by this condensation reaction in the early stage of the sol-gel synthesis process. Thereafter, as the sol-gel synthesis progresses, the silica seed particles formed on the surface of the core particles grow and the new seed particles are generated at the vacant sites on the surface, simultaneously. Furthermore, as the sol-gel synthesis progresses, relatively large silica particles form in the early stage of sol-gel synthesis and relatively small silica particles form in the late stage of sol-gel synthesis.

For the reaction in the early stage of sol-gel (approximately 15 min), small silica particles (approximately 20 nm) as seed particles are observed, although most of the silica particles are very small (Fig. 6B and C). As the sol-gel process proceeds for 30 min, the number of silica particles increases dramatically, and the size of silica particles increased to over 40 nm, simultaneously. Since this stage is an intermediate state of the sol-gel synthesis, the growth of the silica nanoparticles produces mostly irregular shapes and sizes, and some vacant areas on the surface are observed (Fig. 6D). These results suggest that the formation of silica particles during the early stage of sol-gel synthesis is the generation of seeds on the surface of the polymeric core particles. Simultaneously, the formation and growth of silica seeds progresses. As the reaction proceeds, silica nanoparticles on the surface increase in size to over 60 nm and have a quite regular shape (Fig. 6E). The silica seed particles that formed on the surface of the core particles grow, and new seed particles form at the vacant sites of the surface. Finally, the silica nanoparticles cover the core particles and become completely interconnected. More detailed SEM observations at each reaction time are shown in Fig. S3.

Based on the above experimental results, a simple model for the formation of shells is explained in Fig. 6A. In our experimental condition, the polymeric core particles containing siloxane groups attract the formed nuclei, and the attached nuclei consume the monomers in the medium to form a silica shell on the surface of the core particles. Because there is no surfactant involved in the silica-coating process, the attached nuclei can grow continuously and coalesce with other attached nuclei, forming the compact shell. Simultaneously, in contrast to the previous route, the molecules of the TEOS monomer or oligomer are diffused and adsorbed onto the surface of the core particles. They are subsequently polymerized by condensation (silica polymerization on the surface of core particle), forming free silica nanoparticles from the growth of nuclei (Fig. 6). The present result shows that the well-structured core-shell particles have a shell thickness in the range of 100 nm with 92 µm core particles.

## Conclusion

We have presented an interesting and combined microfluidic method for the synthesis of highly monodisperse organic-inorganic hybrid core-shell particles. This method combines the *in situ* microfluidic preparation of highly monodisperse polymeric core microparticles and the growth of silica nanoparticles as an inorganic shell layer for the synthesis of well-defined monodisperse hierarchical organic-inorganic hybrid particles. It is a much simpler and straightforward approach than other conventional synthetic methods to produce highly monodisperse hybrid particles with controllable size in the micrometer range. The methodology presented here executes the groundwork for highly monodisperse hybrid particles. Furthermore, depending on the physicochemical properties of the precursor materials and the fabrication process, there is strong potential for such materials to be used silica microcapsules utilizing the core as a sacrificial template and controlling the thickness of the silica nanoparticles for the application of drug delivery systems with highly controlled diffusion of the active compound. To achieve effectiveness in broader applications, more studies are required (e.g., control of the shell thickness via multiple additions of reactant, use of other core materials, additional special additives that will not change the shell structure and morphology, etc.). However, we believe that the insights gained from the present study will contribute to increased fabrication and innovation.

## Methods

### Materials

Sylgard 184 poly(dimethylsiloxane) (PDMS) prepolymer was purchased from Dow Corning (Midland, MI, USA). SU-8 3050 photoresist was purchased from Microchem (Newton, MA, USA). Absolute grade ethanol, tetraethyl orthosilicate (TEOS, 98%), 3-(trimethoxysilyl)propyl methacrylate (TPM, 98%), poly(vinyl alcohol) (PVA, 87–89%) and 2-hydroxy-2-methylpropiophenone (Darocur 1173) were purchased from Sigma-Aldrich (St. Louis, MO, USA). Ammonium hydroxide 25% solution in water (NH_4_OH) was obtained from Acros Organics (Morris Plains, NJ, USA). 1,10-Decanediol dimethacrylate (DDMA) was purchased from Polyscience (Warrington, PA, USA). Deionized water (>18 k ohm) was obtained using Milli-Q water. All raw materials were used without further purification.

### Fabrication of microfluidic device

Conventional photolithography process using SU-8 3050 photoresist produced the master mold. A PDMS mixture consisting of the prepolymer and the curing agent (10:1 mass ratio) was mixed with a magnetic stirrer and degassed using a vacuum pump at room temperature. The degassed mixture was poured over the master mold and thermally polymerized at 65 °C for 12 h in a convection oven. The prepared PDMS replica was removed easily from the master mold without damaging the microstructure. The microfluidic device is connected to inlet and outlet ports produced by hole punching with a biopsy punch. The punched PDMS replica and bare PDMS plate were treated with oxygen plasma for 90 s. Finally, the replica was bonded with a bare PDMS plate. The dimensions of the final fabricated microfluidic channels are 50 μm × 100 μm (width × height) for the injection and 200 μm × 100 μm (width × height) for the main reaction channel.

### Generation of monodisperse polymeric core particles

The dispersed phase was a solution prepared by mixing TPM and DDMA in a volume ratio of 7:3, and Darocur 1173 as a photoinitiator was added to this mixture at 5 vol.%. The continuous phase was a 4 wt.% PVA aqueous solution. All solutions were loaded through a Tygon tube connected to the microfluidic device with a flow-focusing channel. For the generation of spherical and monodisperse droplets, the flow rate of both the dispersed phase and continuous phase was controlled by a syringe pump (Harvard Apparatus, PHD 2000 series). The generated droplets were photopolymerized by using the focused UV light (100 W HBO mercury lamp) in the wavelength range for 330 to 380 nm (UV-2A filter, Nikon). The estimated UV exposure time of the droplets in the microchannel was less than 1 sec. The polymerized particles were collected in the reservoir. To remove the continuous phase (PVA solution), the polymerized particles were thoroughly washed three times with ethanol and two times with water. Finally, the prepared polymeric core particles were obtained by drying at 70 °C for 12 h.

### Synthesis of organic-inorganic hybrid particles

To synthesize the organic-inorganic hybrid particles, a sol-gel process was carried out using TEOS as a silica source and an ammonia solution as a sol-gel catalyst. First, polymeric core particles were dispersed in 8.65 ml of ethanol with magnetic stirring. The hydrolysis of silicon ethoxide, which was located on the surface of the prepared polymeric core particles, was carried out using an ammonium hydroxide solution. Next, 1.35 ml of the ammonium hydroxide solution was mixed with the dispersed polymeric core particle solution. After further dispersion using an ultrasonic bath for 5 min, the mixture was stirred for 30 min using a magnetic stirrer. The silica source solution as a precursor for the sol-gel reaction was prepared by mixing 500 μl of TEOS and 9.5 ml of ethanol. The prepared silica source solution was added dropwise to the polymer core particles solution. The reaction mixture was fully sealed and stirred at 300 rpm using a magnetic stirrer for 90 min at room temperature. To remove, residual TEOS and ammonium hydroxide, the resultant particles were washed three times with ethanol. Finally, the organic-inorganic hybrid particles were obtained by centrifugation and drying.

### Characterization

Optical microscopic images of the microfluidic procedure and prepared particles were obtained by using an inverted optical microscope (Eclipse TE2000-U, Nikon, Japan) with a CCD camera (Coolsnap, Roper Scientific, USA). The images were analyzed using an Image-Pro Plus (Media Cybernetics, CA, USA) and ImageJ (http://imagej.nih.gov/ij/) software. The synthesis of microparticles and chemical status of monomers (TPM and DDMA) used in this study were confirmed by several analytical apparatus; an analysis of Fourier transform-infrared spectroscopy (FT-IR) equipped with ATR module were performed using a Bruker Alpha FTIR spectrometer (Bruker Optics GmbH, Ettlingen, Germany). The spectral data was collected at 1.4 cm^−1^ resolution with 64 scans at room temperature. The morphologies of each particle were analyzed by cold-type field-emission scanning electron microscopy (S-4800, Hitachi, Japan) primarily used at low voltage after coating with metal using a Quorumtech K575X sputter coater. A 2 nm osmium coating was applied to the sample as measured using a film thickness monitor. We use a field-emission transmission electron microscope (FE-TEM) (JEM 2100F, JEOL, Japan) equipped with energy-dispersive X-ray spectroscopy (EDS) at an accelerating voltage of 100 keV and an AMT XR41-B 4-megapixel bottom mount CCD camera. The camera’s finite-conjugate optical coupler provides high resolution and flat focus with less than 0.1% distortion for high magnification. The analysis of X-ray photoelectron spectroscopy (XPS) (Multilab 2000 XPS, Thermo Fisher Scientific, USA) fitted with the high-performance Alpha110 hemispherical electron energy analyzer producing high photonic energies from 0.1 to 3 keV provides clear information on the local bonding to understand the surface chemistry of particles and the characterization of silica layer. To observe the cross section of the polymeric core particles, the organic-inorganic hybrid particles were cut into 70 nm ultrathin slices using an ultramicrotome (UCT microtome, Leica, Austria) equipped with a diamond knife (DiATOME), and they were observed using a field-emission transmission electron microscopy (JEM 2100F, JEOL, Japan).

### Data availability

All data generated or analyzed during this study are included in this published article.

## Electronic supplementary material

Microfluidic preparation of monodisperse polymeric microparticles coated with silica nanoparticles

## References

[1] Hayes R, Ahmed A, Edge T, Zhang H (2014). Core-shell particles: preparation, fundamentals and applications in high performance liquid chromatography. J. Chromatogr. A.

[2] Shi J (2014). Design and synthesis of organic-inorganic hybrid capsules for biotechnological applications. Chem. Soc. Rev..

[3] Wang R (2013). Functional protein-organic/inorganic hybrid nanomaterials. Wiley Interdiscip Rev.-Nanomed Nanobiotechnol.

[4] Ai Z, Sun G, Zhou Q, Xie C (2006). Polyacrylate‐core/TiO_2_‐shell nanocomposite particles prepared by *in situ* emulsion polymerization. J. Appl. Polym. Sci..

[5] Yin J (2003). Preparation of PS/TiO_2_ core-shell microspheres and TiO_2_ hollow shells. J. Mat. Sci..

[6] Liu L (2016). Hollow carbon nanosphere embedded with ultrafine Fe_3_O_4_ nanoparticles as high performance Li-ion battery anode. Electrochim. Acta.

[7] Yang Y, Chu Y, Zhang Y, Yang F, Liu J (2006). Polystyrene-ZnO core-shell microspheres and hollow ZnO structures synthesized with the sulfonated polystyrene templates. J. Solid State Chem..

[8] Haraguchi K, Ebato M, Takehisa T (2006). Polymer–clay nanocomposites exhibiting abnormal necking phenomena accompanied by extremely large reversible elongations and excellent transparency. Adv. Mater..

[9] Zhang C (2012). Controllable fabrication of PS/Ag core-shell-shaped nanostructures. Nanoscale Res. Lett..

[10] Cai W, Wang W, Yang Y, Ren G, Chen T (2014). Sulfonated polystyrene spheres as template for fabricating hollow compact silver spheres via silver–mirror reaction at low temperature. RSC Adv..

[11] Kanahara M, Shimomura M, Yabu H (2014). Fabrication of gold nanoparticle–polymer composite particles with raspberry, core–shell and amorphous morphologies at room temperature via electrostatic interactions and diffusion. Soft Matter.

[12] Atre AC (2015). Nanoscale optical tomography with cathodoluminescence spectroscopy. Nat. Nanotechnol..

[13] Ribeiro T, Baleizao C, Farinha JPS (2014). Functional Films from Silica/Polymer Nanoparticles. Materials.

[14] Moghal J, Reid S, Hagerty L, Gardener M, Wakefield G (2013). Development of single layer nanoparticle anti-reflection coating for polymer substrates. Thin Solid Films.

[15] Mebert AM (2016). Silica core–shell particles for the dual delivery of gentamicin and rifamycin antibiotics. J. Mat. Chem. B.

[16] Barthlott W, Neinhuis C (1997). Purity of the sacred lotus, or escape from contamination in biological surfaces. Planta.

[17] Feng L (2002). Super-hydrophobic surfaces: From natural to artificial. Adv. Mater..

[18] Kang SM (2014). A Rapid One-Step Fabrication of Patternable Superhydrophobic Surfaces Driven by Marangoni Instability. Langmuir.

[19] Zhang YL, Xia H, Kim E, Sun HB (2012). Recent developments in superhydrophobic surfaces with unique structural and functional properties. Soft Matter.

[20] Wacker JB, Lignos I, Parashar VK, Gijs MAM (2012). Controlled synthesis of fluorescent silica nanoparticles inside microfluidic droplets. Lab Chip.

[21] Li D, Zhu Y, Mao CB (2013). One-pot synthesis of surface roughness controlled hollow silica spheres with enhanced drug loading and release profiles under ambient conditions in aqueous solutions. J. Mat. Chem. B.

[22] Chen HM (2013). Label-Free Luminescent Mesoporous Silica Nanoparticles for Imaging and Drug Delivery. Theranostics.

[23] Kwon S (2013). Silica-based mesoporous nanoparticles for controlled drug delivery. J. Tissue Eng. Regen. Med..

[24] Yuan L (2011). Preparation of pH-Responsive Mesoporous Silica Nanoparticles and Their Application in Controlled Drug Delivery. J. Phys. Chem. C.

[25] Özbek B, Ünal Ş (2017). Preparation and characterization of polymer-coated mesoporous silica nanoparticles and their application in Subtilisin immobilization. Korean J. Chem. Eng..

[26] Jo BH, Kim CS, Jo YK, Cheong H, Cha HJ (2016). Recent developments and applications of bioinspired silicification. Korean J. Chem. Eng..

[27] Balmer JA (2010). When Does Silica Exchange Occur between Vinyl Polymer– Silica Nanocomposite Particles and Sterically Stabilized Latexes?. Langmuir.

[28] Wang T, Keddie JL (2009). Design and fabrication of colloidal polymer nanocomposites. Adv. Colloid Interface Sci..

[29] Tiarks F, Landfester K, Antonietti M (2001). Silica nanoparticles as surfactants and fillers for latexes made by miniemulsion polymerization. Langmuir.

[30] Castillo SIR (2014). Silica cubes with tunable coating thickness and porosity: From hematite filled silica boxes to hollow silica bubbles. Microporous Mesoporous Mat..

[31] Caruso F, Lichtenfeld H, Giersig M, Möhwald H (1998). Electrostatic self-assembly of silica nanoparticle– polyelectrolyte multilayers on polystyrene latex particles. J. Am. Chem. Soc..

[32] Liu X, He J (2007). Hierarchically structured superhydrophilic coatings fabricated by self-assembling raspberry-like silica nanospheres. J. Colloid Interface Sci..

[33] Dong F, Xie H, Zheng Q, Ha C-S (2017). Superhydrophobic polysilsesquioxane/polystyrene microspheres with controllable morphology: from raspberry-like to flower-like structure. RSC Adv..

[34] Sarma D, Gawlitza K, Rurack K (2016). Polystyrene Core–Silica Shell Particles with Defined Nanoarchitectures as a Versatile Platform for Suspension Array Technology. Langmuir.

[35] Graf C, Vossen DL, Imhof A, van Blaaderen A (2003). A general method to coat colloidal particles with silica. Langmuir.

[36] Cha J, Cui P, Lee J-K (2010). A simple method to synthesize multifunctional silica nanocomposites, NPs@ SiO_2_, using polyvinylpyrrolidone (PVP) as a mediator. J. Mat. Chem..

[37] Oh JK, Drumright R, Siegwart DJ, Matyjaszewski K (2008). The development of microgels/nanogels for drug delivery applications. Prog. Polym. Sci..

[38] Serra CA, Chang Z (2008). Microfluidic‐assisted synthesis of polymer particles. Chem. Eng. Technol..

[39] Xu S (2005). Generation of monodisperse particles by using microfluidics: control over size, shape, and composition. Angew. Chem..

[40] Wang JT, Wang J, Han JJ (2011). Fabrication of advanced particles and particle‐based materials assisted by droplet‐based microfluidics. Small.

[41] Choi CH, Jung JH, Hwang TS, Lee CS (2009). *In Situ* Microfluidic Synthesis of Monodisperse PEG Microspheres. Macromol. Res..

[42] Nam J-O, Choi C-H, Kim J, Kang S-M, Lee C-S (2013). Fabrication of Polymeric Microcapsules in a Microchannel using Formation of Double Emulsion. Korean Chem. Eng. Res..

[43] Nunes, J. K., Tsai, S. S., Wan, J. & Stone, H. A. Dripping and jetting in microfluidic multiphase flows applied to particle and fiber synthesis. *J*. *Phys*. *D-Appl*. *Phys*. **46** (2013). 10.1088/0022-3727/46/11/114002 PMC3634598 23626378

[44] Xu JH, Luo GS, Li SW, Chen GG (2006). Shear force induced monodisperse droplet formation in a microfluidic device by controlling wetting properties. Lab Chip.

[45] Jeong H-H, Noh Y-M, Jang S-C, Lee C-S (2014). Droplet-based Microfluidic Device for High-throughput Screening. Korean Chem. Eng. Res..

[46] Kim C, Park K-S, Kim J, Jeong S-G, Lee C-S (2017). Microfluidic synthesis of monodisperse pectin hydrogel microspheres based on *in situ* gelation and settling collection. J. Chem. Technol. Biotechnol..

[47] Song Y, Lee C-S (2014). *In situ* Gelation of Monodisperse Alginate Hydrogel in Microfluidic Channel Based on Mass Transfer of Calcium Ions. Korean Chem. Eng. Res..

[48] Hackley, V. A. & Ferraris, C. F. *The use of nomenclature in dispersion science and technology*. 3 (U.S. Dept. of Commerce, Technology Administration For sale by the Supt. of Docs., U.S. G.P.O 2001).

[49] Perez LD, Lopez JF, Orozco VH, Kyu T, Lopez BL (2009). Effect of the Chemical Characteristics of Mesoporous Silica MCM-41 on Morphological, Thermal, and Rheological Properties of Composites Based on Polystyrene. J. Appl. Polym. Sci..

[50] Reis AV (2011). Copolymer Hydrogel Microspheres Consisting of Modified Sulfate Chondroitin-co-Poly(N-isopropylacrylamide). J. Appl. Polym. Sci..

[51] Sanaeepur H, Kargari A, Nasernejad B (2014). Aminosilane-functionalization of a nanoporous Y-type zeolite for application in a cellulose acetate based mixed matrix membrane for CO_2_ separation. RSC Adv..

[52] Lou Y, Liu G, Liu S, Shen J, Jin W (2014). A facile way to prepare ceramic-supported graphene oxide composite membrane via silane-graft modification. Appl. Surf. Sci..

[53] Innocenzi P (2003). Infrared spectroscopy of sol–gel derived silica-based films: a spectra-microstructure overview. J. Non-Cryst. Solids.

[54] Chen J, Li Q, Xu R, Xiao F (1996). Distinguishing the Silanol Groups in the Mesoporous Molecular Sieve MCM‐41. Angewandte Chemie International Edition.

[55] Zheng J-Z, Zhou X-P, Xie X-L, Mai Y-W (2010). Silica hybrid particles with nanometre polymer shells and their influence on the toughening of polypropylene. Nanoscale.

[56] Zhang S, Guo M, Chen Z, Liu QH, Liu X (2014). Grafting photosensitive polyurethane onto colloidal silica for use in UV-curing polyurethane nanocomposites. Colloid Surf. A-Physicochem. Eng. Asp..

[57] Darmakkolla SR, Tran H, Gupta A, Rananavare SB (2016). A method to derivatize surface silanol groups to Si-alkyl groups in carbon-doped silicon oxides. RSC Adv..

[58] Liu Z (2015). Novel silicon-doped, silicon and nitrogen-codoped carbon nanomaterials with high activity for the oxygen reduction reaction in alkaline medium. J. Mat. Chem. A.

[59] Min X, Fang M, Liu H, Wu X, Huang Z (2017). Growth, structure, and luminescence properties of novel silica nanowires and interconnected nanorings. Scientific reports.

[60] Masalov VM, Sukhinina NS, Kudrenko EA, Emelchenko GA (2011). Mechanism of formation and nanostructure of Stober silica particles. Nanotechnology.

[61] Du X, Liu XM, Chen HM, He JH (2009). Facile Fabrication of Raspberry-like Composite Nanoparticles and Their Application as Building Blocks for Constructing Superhydrophilic Coatings. J. Phys. Chem. C.

