# Supplementary material for: Microfluidic preparation of monodisperse polymeric microspheres coated with silica nanoparticles
Source: Sci Rep. 2018 Jun 4;8:8525. doi: 10.1038/s41598-018-26829-z (PMC5986865; doi:10.1038/s41598-018-26829-z)
Supplement: Supplementary file 1 — Microfluidic preparation of monodisperse polymeric microparticles coated with silica nanoparticles [file 41598_2018_26829_MOESM1_ESM.pdf]

Supplementary information

Microfluidic preparation of monodisperse polymeric microparticles coated with silica nanoparticles

Dong-Yeong Kim, Si Hyung Jin, Seong-Geun Jeong, Byungjin Lee, Kyoung-Ku Kang and Chang-Soo Lee

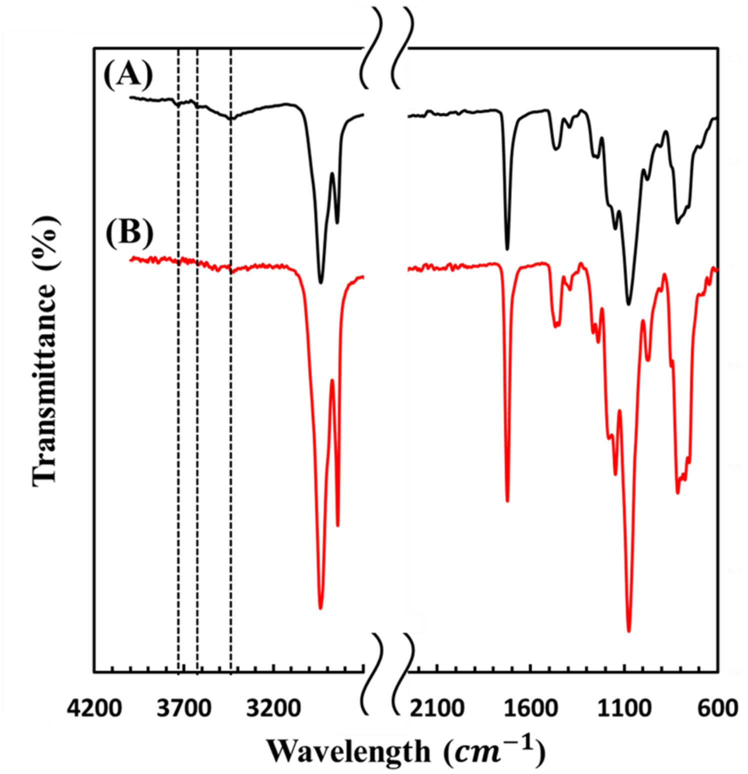

FIGURE S1. FT-IR spectra of organic core particles. (A) after hydrolysis and (B) before hydrolysis process.

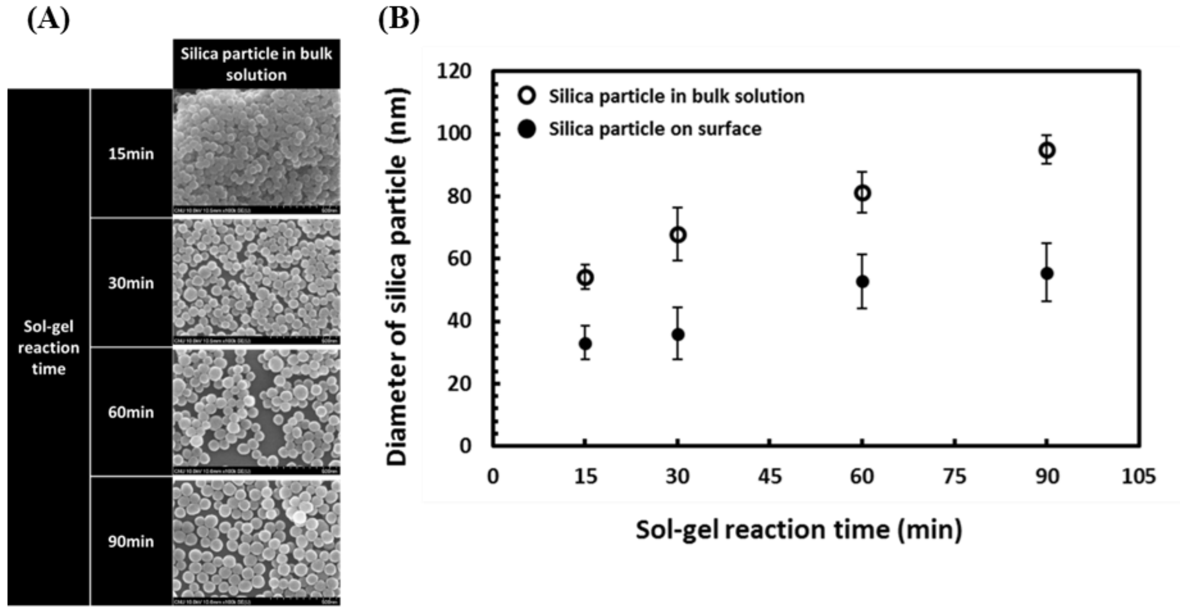

FIGURE S2. Evolution of the particle diameter during sol-gel reaction. (A) The growth of silica particles in bulk solution; this experiments were performed by simple sol-gel reaction in the absence of core polymeric particles. (B) The monitoring of change of the size of silica particles both bulk solution (open circle) and on the surface of the core polymeric particles (closed circle).

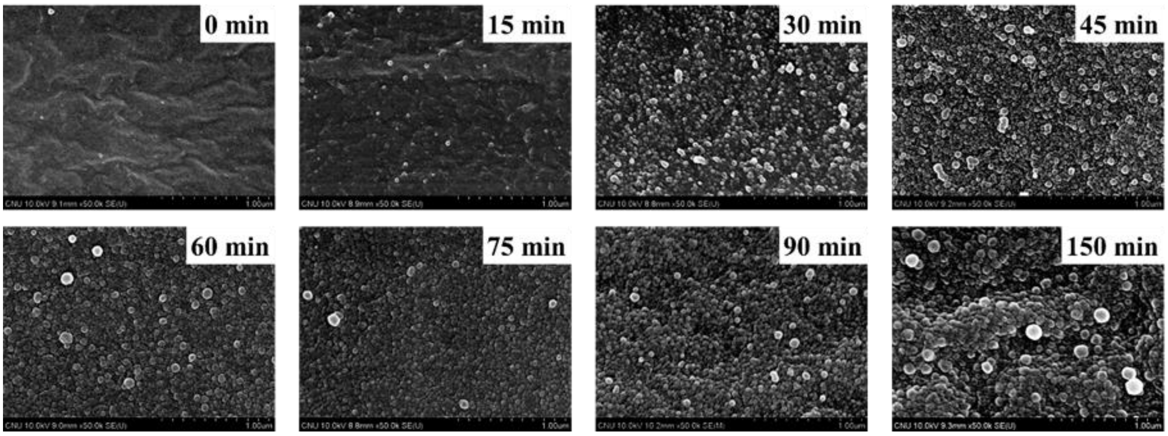

FIGURE S3. SEM images for the analysis of morphological change of silica nanoparticles on the surface of polymeric core particles at each reaction time.
